# The influence of the lack of insulin receptor substrate 2 (IRS2) on the thyroid gland

**DOI:** 10.1038/s41598-019-42198-7

**Published:** 2019-04-05

**Authors:** Maria Carmen Iglesias-Osma, Enrique J. Blanco, Marta Carretero-Hernandez, Leonardo Catalano-Iniesta, Virginia Sanchez-Robledo, Maria Jose Garcia-Barrado, Teresa Vicente-Garcia, Deborah J. Burks, Jose Carretero

**Affiliations:** 1 Department of Physiology and Pharmacology, Faculty of Medicine, University of Salamanca, Salamanca, Spain; 2 Laboratory of Neuroendocrinology, Institute of Neurosciences of Castilla y León (INCyL), and Laboratory of Neuroendocrinology and Obesity, Institute of Biomedical Research of Salamanca (IBSAL), University of Salamanca, Salamanca, Spain; 3 Department of Human Anatomy and Histology, Faculty of Medicine, University of Salamanca, Salamanca, Spain; 4 Laboratory of Molecular Neuroendocrinology, Principe Felipe Research Center (CIPF), Valencia, Spain

## Abstract

Involvement of IRS2 in the proliferative effects of IGF-I of follicular thyroid cells has been described, but there are no evidences for *in vivo* participation of IRS2. This study aimed to analyse the *in vivo* relevance of IRS2 in the proliferation and apoptosis of thyroid cells by immunocytochemical studies for PCNA, Ki67, and active-caspase-3 in thyroid cells of IRS2 knockout (IRS2-KO) mice, jointly to TUNEL assay. Thyroid hormones were lower in IRS2-KO mice than in their wild-type (WT) counterparts. Increases in the area, perimeter and diameter of thyroid follicles of IRS2-KO mice were observed, which also showed increased proliferation rate of follicular cells and decreased percentage of apoptotic cells that was more evident in the central than in the marginal region of the gland. Sex-related differences were also found, since the follicular epithelium height was higher in male than in female mice. The percentage of proliferating cells showed significant changes in male but not in female mice, and apoptotic cells were more abundant in female than in male IRS2-KO animals, without significant differences between WT-animals. Therefore, our results suggest that IRS2 could be involved in the maintenance of thyroid cells population and in the normal physiology of the thyroid gland.

## Introduction

*In vitro*, IGF-I (insulin-like growth factor-I) and TSH (thyroid-stimulating hormone) could act synergistically on proliferation of follicular thyroid cells^1,2^ and their mediation on proliferation and differentiation in thyroid cells is interdependent^3–5^. IGF-I (or insulin at supraphysiological concentrations) is required for the mitogenic action of TSH, whose effects on thyroid gland growth are mediated by the adenylate cyclase-cAMP cascade. Moreover, TSH stimulates thyroid follicular cell proliferation, differentiation, and function through the cAMP-signalling pathway^6^.

The physiological actions of IGF-I and insulin are mediated via the tyrosine phosphorylation of insulin receptor substrate (IRS) proteins, mainly IRS1 and IRS2. The IRSs are important intracellular intermediaries of insulin and IGF1 effects. So, when IGF-I binds to the IGF-I receptor, the receptor intrinsic tyrosine kinase is activated and phosphorylates intracellular substrates including IRS1 and IRS2. Once phosphorylated, IRS proteins recruit other signalling complexes, such as phosphatidylinositol-3-kinase (PI 3-kinase), to orchestrate the cellular response to insulin/IGF-I binding^7^.

*In vitro* IGF-I-induced phosphorylation of IRS2 is enhanced by pre-treatment with cAMP without causing changes in the total amount of IRS2. Changes in phosphorylation of IRS2 induce an increase of total PI 3-kinase activity, which is necessary for the proliferative effects of IGF-I^8^. Moreover, high circulating levels of insulin associated with resistance to this hormone may increase thyroid cell proliferation^9^.

It has been described that during goitrogenesis thyroid IRS1 mRNA expression increases progressively and is associated with the increased rate of cell mitosis promoted by TSH. This suggests that insulin and IGF-I are important *in vivo* co-mitogenic factors that possibly act through the activation of IRS1^10^. However, the *in vitro* proliferative effects of IGF-I after pre-treatment with cAMP are accompanied by an increase in the phosphorylation of IRS2, but not of IRS1^8^.

The aim of this study was to analyse the *in vivo* relevance of IRS2 in the proliferation and apoptosis of thyroid follicular cells, because although the *in vitro* participation of IRS2 in the proliferative effects of IGF-I has been well described, there are few studies that have analysed the *in vivo* effects of the lack of IRS2 on proliferation and apoptosis of thyroid follicular cells.

The relationship between goitre and type 2 diabetes mellitus has been reported in some clinical studies, where there are differences that are more pronounced in women than in men. These studies explain the increase in glandular volume caused by the hyperinsulinemia, which develops as a means to compensate for the hyperglycaemic status^9,11–14^. Deletion of IRS2 in mice causes insulin resistance and a profound decrease in ß-cell mass, which culminates in diabetes^15^. In order to avoid hyperglycaemic effects, the present study was carried out in IRS2-KO mice with normal glycaemia.

## Results

### Thyroid gland

**Figure 1:**
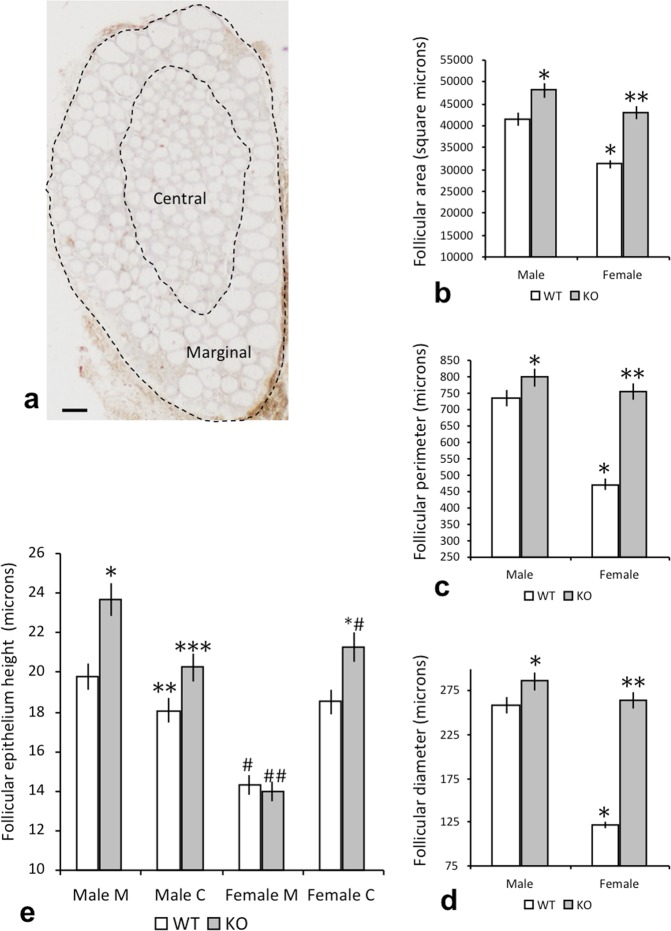
Morphoplanimetry of the thyroid gland in WT and IRS2-KO mice of both sexes. (**a**) Sagittal section of the thyroid gland, obtained from a wild-type male, with a large number of follicles; the central and marginal regions of the gland are delimited by the dotted lines. Scale bar: 120 µm. (**b**–**d**) Gender differences in the parameters of the thyroid follicles of WT and IRS2-KO animals: (**b**) follicular area, (**c**) follicular perimeter, (**d**) follicular diameter. ^*^p < 0.05 in relation to WT males, ^**^p < 0.05 with respect to KO males and p < 0.01 with respect to WT females. (**e**) Differences regarding the height of the follicular epithelium in the central (C) and marginal (M) regions of the gland. ^*^p < 0.05 in relation to the marginal region in WT males, ^**^p < 0.05 with respect to the marginal region in WT males and the central region in KO males, ^***^p < 0.05 in relation to the marginal region of KO males, # p < 0.01 with respect to the central region of WT females and the marginal region of WT males, ## p < 0.01 with respect to the marginal region of KO males, ^*^#p < 0.05 with respect to the central region of WT females.

Figure 1a shows a sagittal section of the thyroid gland, where the central and marginal regions of the gland are delimited using dotted lines and contain a large number of follicles.

Morphometric data related to the measurements of the area, perimeter and diameter of the follicles (Fig. 1b–d) revealed that the size of the thyroid follicles in female mice was smaller than in the males in both the IRS2-KO mice (p < 0.05) and the controls (p < 0.05). In addition, a significant difference was also found between the follicular size of WT and IRS2-KO (p < 0.01) female mice, as the three parameters measured (area, perimeter and diameter) were greater in the latter (Fig. 1b–d).

With respect to the height of the cells of the follicular epithelium, our measurements distinguished between follicles located marginally and those located at the central region of the thyroid gland (Fig. 1e). The data showed that in male mice the follicular height of the marginal region was higher than in the central region (Fig. 1e; p < 0.05), whereas in females the opposite occurred and a significantly greater height was observed in the central follicles (Fig. 1e; p < 0.01).

In all cases, follicular height was greater in the IRS2-KO animals than in WT mice (Fig. 1e), with the exception of the marginal region of both WT and IRS2-KO female animals, where similar values were detected. The height of the follicular epithelium in the marginal section was higher in male mice than in female mice (Fig. 1e; p < 0.01). In contrast, there were no significant differences between males and females with respect to the height of the central follicular epithelium, either in the WT animals or in the IRS2-KO group (Fig. 1e).

### Immunoreactivity for PCNA and Ki67

**Figure 2:**
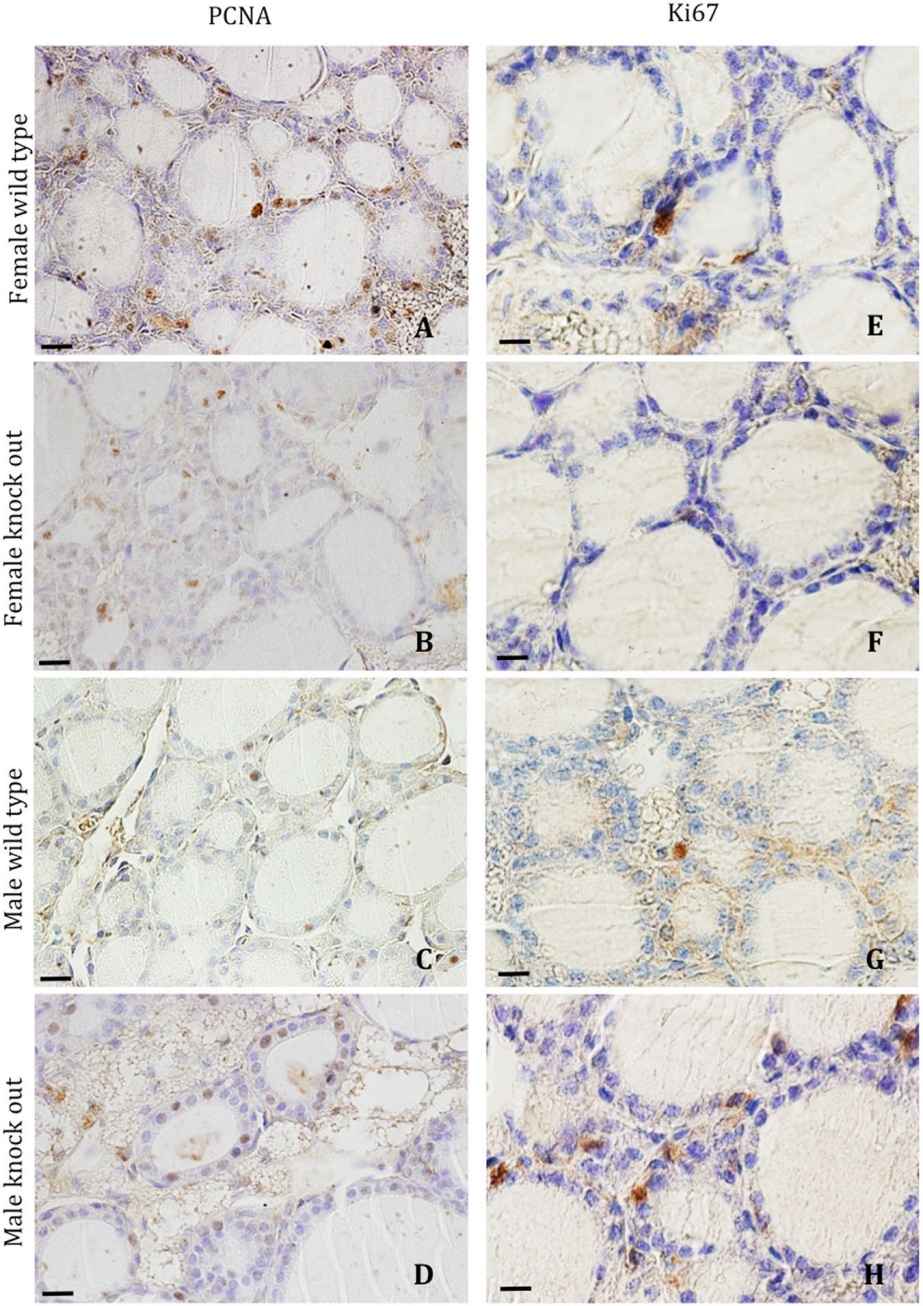
Immunohistochemical reaction for PCNA (**A**–**D**) and Ki67 (**E**–**H**) in thyroid follicular cells. A similar amount of PCNA- and Ki67-positive follicles may be observed in female WT (**A,E**) and IRS2-KO (**B,F**) animals, and a greater amount of positive follicles in male WT (**C,G**) and IRS2-KO (**D,H**) mice. Scale bars: A–D = 30 µm; E–H = 20 µm.

**Figure 3:**
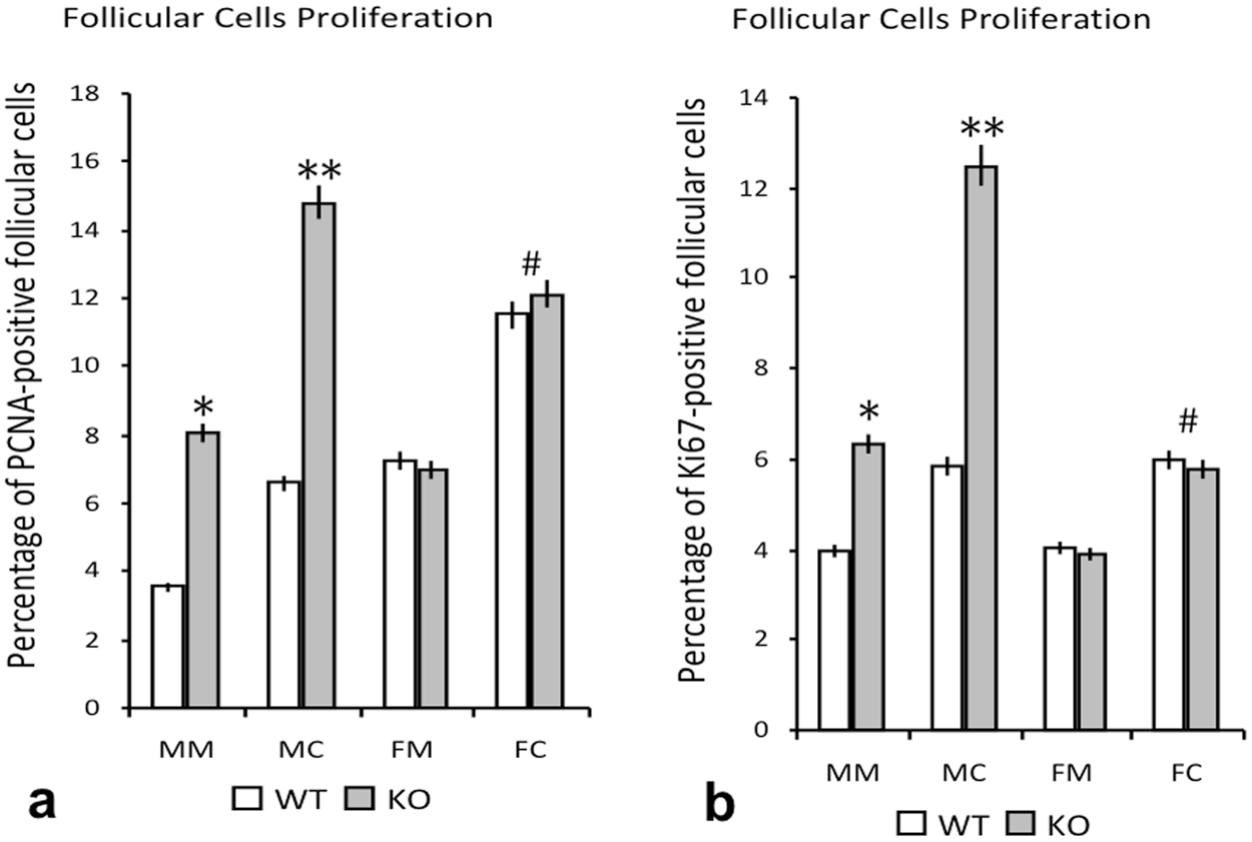
Percentage of proliferation in thyroid follicular cells of WT and IRS2-KO mice of both sexes. The plots show the comparison between wild-type (WT) and IRS2 deficient animals (KO), regarding the gender and regions of the gland (MM: male marginal; MC: male central; FM: female marginal; FC: female central), and the values are expressed as mean ± SEM. (**a**) Graphic showing the percentage of proliferation by PCNA-positive follicular cells. Significant differences are defined as: ^*^ p < 0.01 with respect to WT MM; ^**^p < 0.01 with respect to WT MC and KO MM, and p < 0.05 with respect to KO FC; #p < 0.05 with respect to FM. (**b**) Plot showing the percentage of proliferation by Ki67-positive follicular cells. Statistically different at: ^*^ p < 0.05 with respect to WT MM and KO FM; ^**^p < 0.01 with respect to WT MC, KO MM and KO FC; #p < 0.05 with respect to FM.

The PCNA or Ki67 immunoreactivity was observed mainly in follicular cell nuclei (Fig. 2), both in the central and marginal regions. In WT mice, the percentage of PCNA- or Ki67-positive follicular cells was greater in the central follicular region than in the marginal region (Fig. 3a,b). Although, in both regions, similar values were found for Ki67-positivity between male and female animals, the percentages of PCNA-positive cells were higher in female than in male animals. In male animals, the lack of IRS2 induces a significant increase of the percentages of PCNA- and Ki67-positive follicular cells that was not found in female animals (Fig. 3a,b). These differences were higher in the central regions compared to marginal regions. These increases were more important in male than in female animals.

### Immunoreactivity for active caspase-3 and Terminal deoxynucleotidyl transferase dUTP nick end labelling (TUNEL) assay

**Figure 4:**
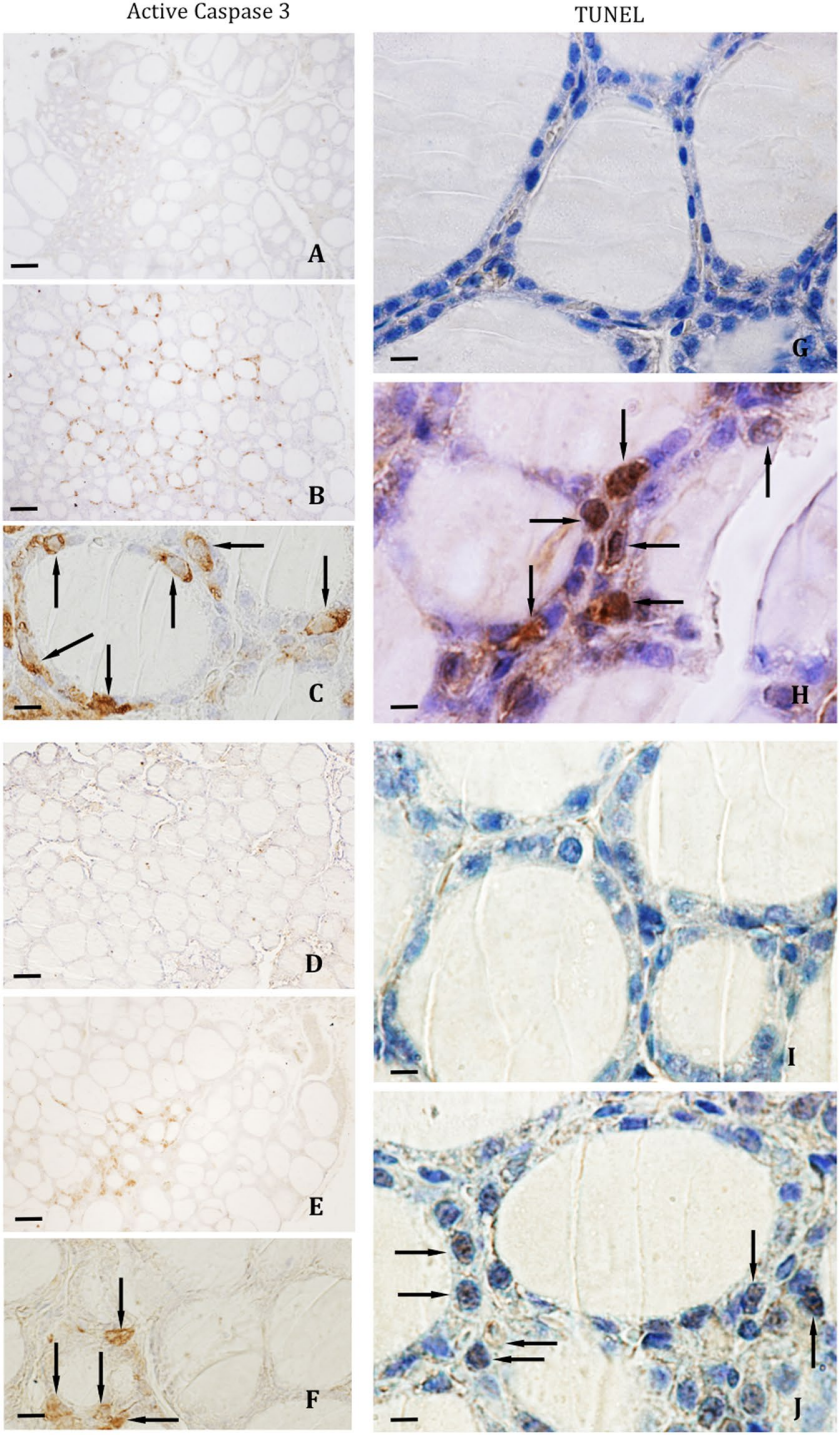
Immunohistochemical reaction for active caspase-3 and TUNEL assay in thyroid follicular cells. Follicular active caspase 3-positive cells in WT female (**A**) and male animals (**D**), and IRS2-KO female (**B–C** arrows) and male (**E**–**F** arrows) mice. TUNEL-positive follicular cells were less numerous in the WT female (**G**) and male (**I**) mice than in the IRS2-KO female (**H**, arrows) and male (**J**, arrows) animals. Scale bars: A, B, D, E = 60 µm; C, F = 20 µm; G–J = 12 µm.

**Figure 5:**
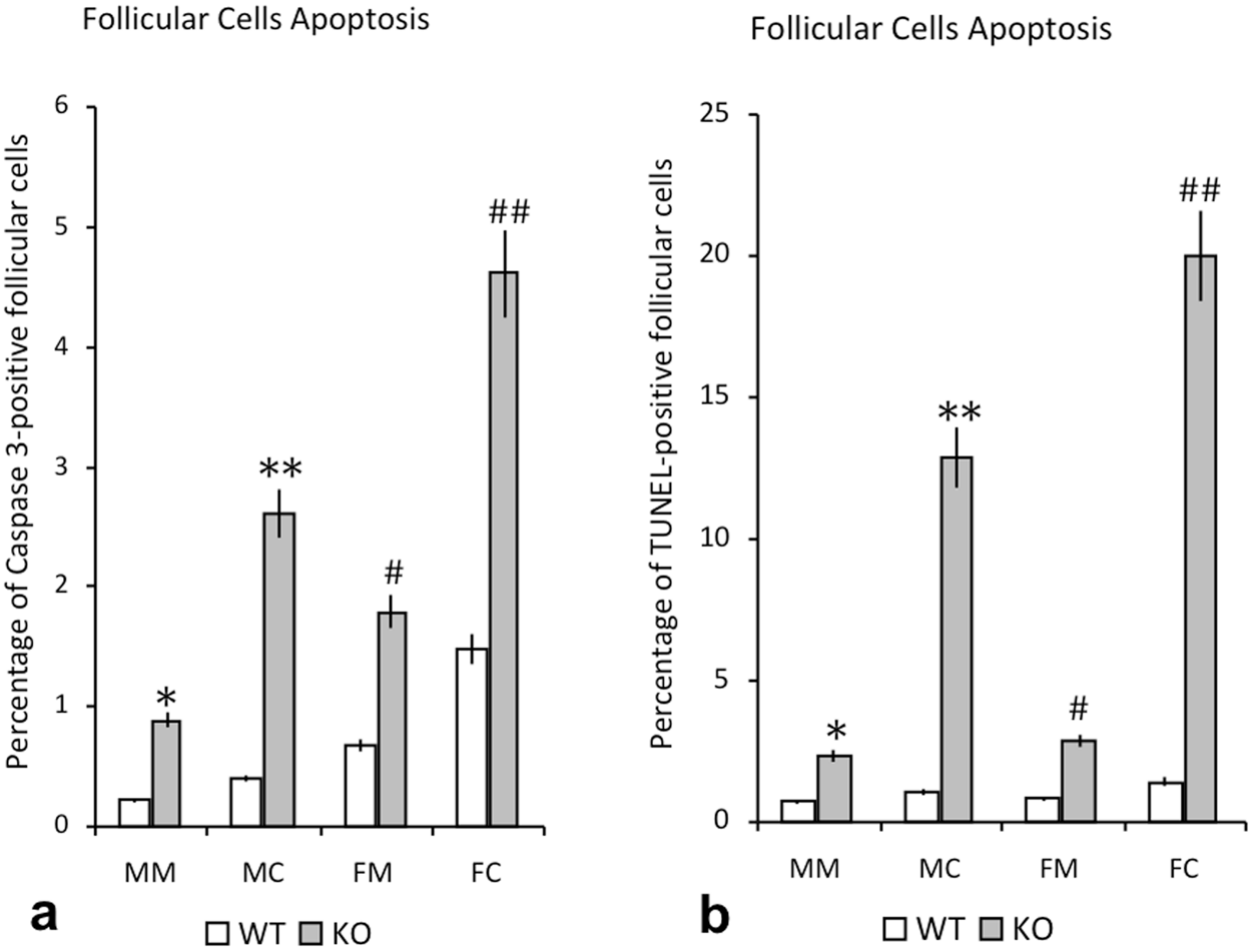
Percentage of apoptosis in thyroid follicular cells of WT and IRS2-KO mice of both sexes. The plots show the comparison between wild-type (WT) and IRS2 deficient animals (KO), regarding the gender and regions of the gland (MM: male marginal; MC: male central; FM: female marginal; FC: female central), and the values are expressed as mean ± SEM. (**a**) Graphic showing the percentage of apoptosis by active caspase-3-positive follicular cells. Significant differences are defined as: ^*^p < 0.01 with respect to WT MM, ^**^p < 0.01 with respect to WT MC and KO MM; #p < 0.01 with respect to WT FM and KO MM; ##p < 0.01 with respect to WT FC, KO FM and KO MC. (**b**) Plot showing the percentage of apoptosis by TUNEL-positive follicular cells. Significant differences are defined as: ^*^p < 0.05 with respect to WT MM; ^**^p < 0.01 with respect to WT MC and KO MM; #p < 0.05 with respect to WT FM; ##p < 0.01 with respect to WT FC and KO FM and p < 0.05 with respect to KO MC.

The active caspase-3 immunoreactivity was observed in the cytoplasm of follicular cells (Fig. 4A–F) and the reaction for TUNEL assay was a nuclear one (Fig. 4G–J). In WT mice, the percentage of active caspase-3- or TUNEL-positive follicular cells was greater in the central follicular region than in the marginal region (Fig. 5a,b). Both in male and female IRS2-KO animals (Fig. 4B,C,E,F,H,J) the percentages were significantly increased in relation to WT mice. These differences were higher in the central regions compared to marginal regions and they were more relevant in female than in male animals (Fig. 5a,b).

### Thyroid hormone levels

**Figure 6:**
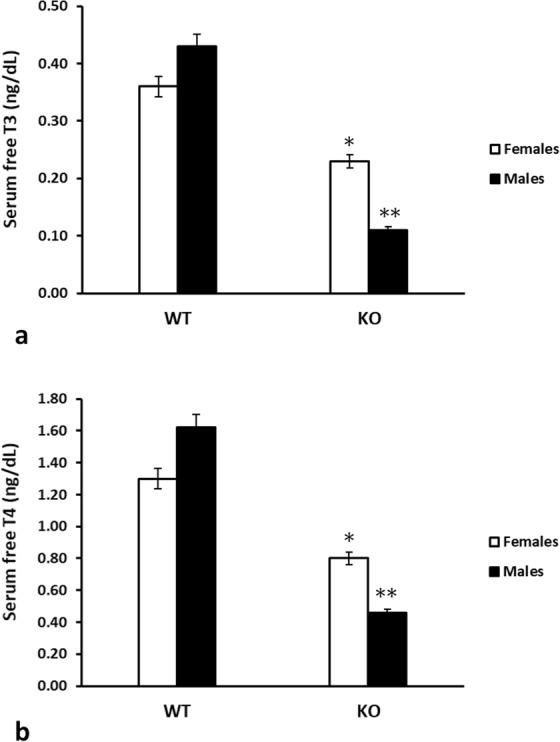
Serum concentrations of thyroid hormones (ng/dL) in WT and IRS2-KO mice of both sexes. The levels of (**a**) free triiodothyronine (FT3) and (**b**) free thyroxine (FT4) are depicted for wild-type (WT) and IRS2 knockout (KO) animals, in female as well as in male mice. ^*^p < 0.05 with respect to WT females; ^**^p < 0.01 with respect to WT males, and p < 0.05 with respect to females animals.

Figure 6 shows the measurements obtained for FT3 (Fig. 6a) and FT4 (Fig. 6b), where the serum concentrations of FT3 and FT4 in WT animals were similar in female and male mice, and were in the euthyroid range. Although FT3 and FT4 were lower in IRS2-KO female mice than in the WT female controls (p < 0.05), the levels were also within the euthyroid range. In male animals, the IRS2-KO mice showed lower levels of both hormones than in the WT (p < 0.01). When comparing the results obtained for both sexes, the male IRS2-KO mice also presented reduced concentrations of both hormones with respect to the female group (p < 0.05) (Fig. 6a,b).

## Discussion

The association of diabetes mellitus with thyroid disorders has been documented in several studies, where thyroid disease is more prevalent in diabetics as compared to nondiabetics and in women as compared to men. Indeed, thyroid dysfunction remains the most frequent autoimmune disorder associated with type 1 diabetes^16^. Moreover, thyroid enlargement has been reported in patients with type 2 diabetes, since hyperinsulinemia induced by insulin resistance may act by increasing thyroid proliferation as a consequence of the mitogenic actions of the hormone^9,11,14^.

This is the first report analysing the morphometric parameters of thyroid follicular cells in non-hyperglycaemic IRS2 knockout mice. As is described in our results, when comparing with the WT animals, in the thyroid follicles of IRS2-KO mice there was observed an increase in the area, perimeter and diameter. Jointly, the IRS2-KO animals showed an increased proliferation rate of the follicular cells, and decreased percentage of apoptotic cells, which was more evident in the central than in the marginal region of the gland. Interestingly, in the IRS2 deficient animals there were also found gender differences since the follicular epithelium height was higher in male than in female mice. Though, we have also previously demonstrated in goiter rodents induced with methimazole, slight gender differences with an increased follicular epithelium height that was higher in female than in male animals^17^. Thus, our findings indicate a dysregulated proliferative reaction in the follicular thyroid cells of the normoglycaemic IRS2 deficient male animals compared to their female counterparts, suggesting that the lack of IRS2 could be responsible for such response.

Generally, it is considered that the flattening of the thyroid follicular epithelium is a finding indicating a low activation of the follicular cells^18,19^. Nonetheless, variations in the surface and diameter of the follicular lumen could have different interpretations because thyroid follicles are heterogeneous, so their functional status is often difficult to be defined^19^. Accordingly, increases of both parameters could also be relating to an increase in the release of thyroglobulin from epithelial cells or to a decreased reuptake of thyroglobulin from the lumen to the cytoplasm of the follicular cells, in order to synthesise thyroid hormones^17^.

Furthermore, we have examined the proliferation and the apoptosis of follicular cells in these animals, by means of the immunocytochemical studies for PCNA, Ki67, and active caspase-3, together to the TUNEL method, by applying a similar approach as used in previous studies of the thyroid morphology carried out by our group^18,20^. As we have previously shown in normal rodent thyroid tissue, total cell mass is maintained through a balance between cell proliferation and apoptosis^18^. Additionally, it has been documented that, in normal thyroid glands, the proliferative activity of the thyrocytes decrease with age and show a low proliferation index in the adulthood^21^. Furthermore, TSH and IGF-I are interdependent in stimulating proliferation in thyroid cells, as well as in regulating cell function, and TSH, which acts through a cAMP-dependent pathway, potentiates DNA synthesis induced by IGF-I^8,22^. In this regard, it is known that TSH and IGF-I are essential for thyrocyte proliferation and act synergically in the stimulation of thyroid cell growth, which occurs by increasing the intracellular levels of cAMP and activating proliferation pathways including the phosphatidylinositol 3-kinase (PI3K)-Akt pathways^23^. In fact, it has been suggested that the activation of adenylate cyclase is sufficient to stimulate thyroid cell growth^24^. Moreover, high circulating levels of insulin associated with resistance to this hormone may also increase thyroid proliferation^9,13^.

Our current results demonstrate that the percentage of proliferating PCNA-positive cells, as well as Ki67-positive cells, displayed significant changes in the male IRS2-KO animals but not in female IRS2 deficient mice. IRS proteins are key mediators in insulin and IGF-I signalling, and even though IRS family members possess a similar structure, the tyrosine phosphorylation of each IRS is regulated in a distinct manner, suggesting that each IRS has a different physiological role^25,26^. Deletion of IRS2 produces diabetes in mice, owing to peripheral insulin resistance (in liver and adipose tissue, principally) and a reduction in pancreatic ß-cell mass^15^. IRS2 also plays a role in the development and growth of several tissues and organs (as the pancreatic beta-cells, testis, muscle or brain), potentially by mediating IGF-I signalling via the Protein kinase B or Akt (PKB/Akt) and AMP-activated protein kinase (AMPK) pathways, which could be critically affected by dysregulation of the IRS2 branch of the insulin-IGF-signalling cascade^27–30^. Certainly, the metabolic alterations, which underlie the sexual dimorphism described for the diabetic phenotype of Irs2^−/−^ mice, appear to be associated with differential defects in the cAMP system. This includes impaired generation of cAMP in the adipose tissue of female Irs2^−/−^ mice and an increased sensitivity to cAMP-generation in islets and adipocytes of male Irs2^−/−^ mice, with reduced expression of alpha 2-adrenoceptors (α2-AR) in ß-cells^31^.

Indeed, the insulin resistance and hyperinsulinemia can increase cellular proliferation by different mechanisms: activation of the mitogen-activated protein kinase (MAPK) pathway (to overcome the inhibition and restore the PI3K pathway), higher synthesis of bioactive IGF-I, and the binding of insulin to IGF-binding proteins (IGFBPs), with the displacement and augmented levels of free IGF^14,32^. Thus, the disruption of the IRS2 gene and the consequent dysregulated insulin secretion and hyperinsulinemia described in the male Irs2^−/−^ mice joint to the increase in intracellular cAMP levels linked to a sexual dimorphism^31^, could underlie in proliferation of thyrocytes and other morphological findings observed in the present study in the thyroid follicles of those male IRS2-KO animals. Sex-specific differences associated with thyroid function have been reported in male and female mice of some traits, but are related to genetic linkage^33^.

Respect to the study of apoptosis, our present data show that the percentage of active caspase-3-positive cells were significantly increased in both sexes of IRS2 deficient mice, being more abundant in the female IRS2-KO mice than in the male animals lacking Irs2, which was also corroborated by TUNEL assay. Interestingly, our results are in agreement with other reports, which analysed the apoptosis of the chromaffin cells in the adrenal medulla by studying the immunoreactivity for active caspase-3, observing in both genders of IRS2-KO mice a significant increase in the percentage of active caspase-3-positive chromaffin cells, with an increment that was more relevant in the female Irs2^−/−^ animals^34^.

Regarding the thyroid hormones levels, the serum concentrations of FT3 and FT4 were lower in IRS2-KO mice than in their WT counterparts, and the male IRS2-KO mice also presented reduced concentrations of both hormones when comparing to the female group. It is worth noting that these results could be relating to the sexual dimorphism described for this diabetic animal model that depends on metabolic and hormonal variations, which are linked to differential defects in the cAMP signalling system, as detailed above.

In decompensated diabetics, where reduced T3 levels have been observed, this “low T3 state” has been explained by an impairment in the peripheral conversion of T4 to T3, which normalizes when glycaemic control improves^16^. However, in our study, the IRS2 deficient mice were normoglycaemic and the reduced T3 levels were accompanied by low levels of FT4, where glycaemic levels upon fasting and after feeding were similar to those of the WT control animals. These findings suggest that the deletion of IRS2, more than the glycaemic levels as such, is responsible for hormonal decline.

Remarkably, the female IRS2 knockout mice develop moderate obesity with hypothalamic leptin resistance and hyperleptinemia^35^, and gender differences with respect to adiponectin plasmatic levels have also been reported^36^. Therefore, it could be suggested that in IRS2-deficient obese female mice, the mitogenic actions of leptin (and other adipocyte-released factors) point to an excess of oestrogens (ovarian and derived from peripheral aromatization in the adipose tissue), which could explain the smaller size of their follicular cells and the scarce thyroid proliferation observed in the female Irs2^−/−^ mice.

This is the first study describing the morphological characteristics of the thyroid follicles in Irs2^−/−^ mice. In addition, it is worth noting that significant proliferation of the thyrocytes was observed in the IRS2-deficient mice of both sexes. Considering the cross-talk among several pathways, and the hormones and the cytokines implicated in the IRS2 null mice (i.e., hyperinsulinemia in male, and adipocyte-derived hormones as leptin or oestrogens in female), the findings observed in the thyroid follicles of this diabetic phenotype could most likely be multifactorial.

In summary, our results suggest that IRS2 could be directly or indirectly involved, as well as its intracellular signalling cascade activated after IRS2 phosphorylation, in the maintenance of thyroid cells thus contributing to the physiologic processes of the thyroid gland.

## Materials and Methods

### Animals and experimental protocol

The experiments were carried out following the protocols and ethical requirements approved by the Committee for the Care and Use of Animals of the University of Salamanca, in accordance with the regulations for the use of animals in research studies established by the EU Directive (2010/63/EU) and current Spanish legislation (RD 53/2013).

The generation of IRS2-deficient mice and the genotyping of the animals using PCR have been previously described^37^. The mice used in the present study were maintained in a C57BL/6 background and WT littermates were used as the controls. Adult Irs2^−/−^mice (8–10 weeks old) and age-matched WT mice of both sexes were used in this study, which included 5 mice per strain, age and sex. All mice were maintained under controlled conditions, in a room at 21 ± 2 °C, with 50 ± 5% relative humidity, a light-dark cycle of 12/12 h and were allowed free access to food (irradiated chow, Harlan 20/14) and water.

In order to examine the consequences of IRS2 deficiency under conditions that minimized the complications of diabetic metabolism, the glycaemic levels were routinely determined by extracting a small quantity of blood from the tail for its immediate analysis using a glucometer (Bayer Elite® model, Bayer Healthcare, Barcelona, Spain). The mice which were overtly diabetic (fasting glycaemia >120 mg/dL or fed glycaemia >220 mg/dL) were excluded from the study.

### Sacrifice of animals and sample processing

Animals were sacrificed by decapitation under isoflurane-anaesthesia and the thyroid glands were obtained through careful dissection. The glands were fixed by immersion in 4% paraformaldehyde in phosphate buffer (0.1 N, pH 7.4), dehydrated in ethanol, cleared in xylene and embedded in paraffin. Serial sections of 5 µm thickness were obtained and mounted on slides for immunocytochemical study.

### Immunocytochemistry

A streptavidin-peroxidase immunocytochemical method was developed for studying the Proliferating Cell Nuclear Antigen (PCNA), Ki67, and caspase-3-positive cells.

After blocking endogenous peroxidase with 0.5% H_2_O_2_ in methanol and non-specific reactions of the secondary antibody by incubation in normal goat serum (Dako®, 1:30) in TBS (Tris-saline buffer 0.05 M, pH 7.6 in 0.8% NaCl), the samples were incubated overnight at 4 °C with the mouse serum anti-PCNA IgG (Dako®, diluted 1:2000 in TBS), rabbit polyclonal serum anti-Ki67 (Abcam®, ab15580; diluted 1:700 in TBS), and rabbit serum anti-active caspase-3 (Sigma®, Sigma-Aldrich Inc., St. Louis, MO, USA, diluted 1:1000 in TBS). Biotinylated goat anti-mouse IgG (Dako®, diluted 1:150) or biotinylated goat-anti-rabbit IgG (Dako®, diluted 1:150 in TBS) and streptavidin-peroxidase complex (Dako®, 1:150) were successively applied at room temperature for 40 minutes. The reaction was developed in freshly prepared 0.25% 3,3′-diaminobenzidine tetra hydrochloride (DAB; Sigma®, Sigma-Aldrich Inc., St. Louis, MO, USA) in TRIS buffer containing 0.03% H_2_O_2_.

For Ki67 immunodetection, the samples were pre-treated using heat mediated antigen retrieval with sodium citrate buffer (pH 6) for 20 min.

Controls included substitution of the primary antibodies with normal, mouse or rabbit, serum or TBS, and omission of the secondary antibody or streptavidin-conjugated peroxidase (Stav-Pox) complex. No immunoreactivity was detected after carrying out these tests.

### Detection of DNA fragmentation by TUNEL method

*In situ* apoptosis detection was carried out by using a commercially available Terminal deoxynucleotidyl transferase dUTP nick end labelling (TUNEL) apoptosis kit (Abcam®, ab206386; Abcam Biotechnology, Cambridge, UK). Following the manufacturer’s instructions, in this colorimetric assay Terminal deoxynucleotidyl Transferase (TdT) binds to exposed 3′-OH ends of DNA fragments generated in response to apoptotic signals and catalyses the addition of biotin-labelled deoxynucleotides. Biotinylated nucleotides were detected using a streptavidin-horseradish peroxidase (HRP) conjugate. DAB reacted with the HRP labelled sample to generate an insoluble brown coloured substrate at the site of DNA fragmentation. Subsequently, the samples were counterstained with Mayer’s hematoxylin for the morphological evaluation and characterization of normal and apoptotic cells.

### Morphoplanimetry

Cell counts were performed from digital microphotographs obtained by using a Zeiss® Axiophot microscope with an Olympus® digital camera. Four hundred thyroid follicular cells per region were randomly selected from 10 sections of the thyroid gland of each animal, with a distance of 50 µm between two groups of cells: 200 cells from the central part and 200 cells from the marginal part of the sections. From these cells, the number of caspase-3-positive, PCNA-positive, TUNEL-positive, and Ki67-positive thyroid follicular cells were counted (only nuclear TUNEL, Ki67 or PCNA expression was considered, except in metaphasic-mitotic cells), and were calculated as a percentage of the total number of cells analysed.

Using the ImageJ software (National Institutes of Health, USA), 100 thyroid follicles per animal were analysed. The global follicular area, global follicular perimeter and Ferret’s follicular diameter were determined by following the external limits surrounding the follicles. In addition, the distance from the luminal to basal cell membranes was measured in order to determine the follicular epithelium height for each follicle.

### Quantification of thyroid hormones

Immediately after the mice were sacrificed, blood was collected from trunk blood after decapitation and the serum was obtained by centrifugation at 1000 × *g* for 20 minutes and stored at −20 °C until assayed. Serum free triiodothyronine (FT3) and free thyroxine (FT4) concentrations were measured with an automated analyser using a commercially available electrochemiluminescence immunoassay (Roche Diagnostics GmbH, Mannheim, Germany), following the manufacturer’s instructions. The intervals of detectable concentrations provided by the manufacturer were 0.4–50 pmol/L for FT3 and 0.3–100 pmol/L for FT4. All samples were run in duplicate in a single assay for each hormone, and hormonal concentrations were calculated using a standard kit, where the FT3 and FT4 values were recorded as ng/dL.

### Statistical analysis

The values were analysed statistically using GraphPad Prism 6 (GraphPad Software Inc., La Jolla, CA, USA) and were subjected to ANOVA followed by the Scheffé F test for multiple comparisons. The values were considered significant when p < 0.05. The results are expressed as mean values ± SEM (standard error of the mean).
